# Tuberculosis - The spoil sport

**DOI:** 10.4103/0971-3026.41843

**Published:** 2008-08

**Authors:** MJ Govindarajan, Nagaraj, Ravikumar, Arjun Kalyanpur, Kallur

**Affiliations:** Bangalore Institute of Oncology- Health Care Global-Bangalore, Teleradiology Solutions, Bangalore, India. E-mail: revathigovind@gmail.com

Dear Sir,

This is in response to the article ‘Pictorial essay: PET/CT in tuberculosis’ by Harkirat et al. We could not agree more with the opinion of the authors. When PET/CT is drawing so much attention from the whole medical fraternity, particularly due to its very high sensitivity in cancer staging and re-staging, a very common and old disease called tuberculosis is spoiling the party, especially so in countries like India where the prevalence of tuberculosis is significantly higher than in the West. Tuberculosis frequently masquerades as malignancy and, when extensive, is often mistaken for the same. We wish to share with our colleagues our experience with two such cases, where the clinical suspicion of tuberculosis was not high although the patients were later found to have extensive disease throughout the body.

## Case 1

**Figure 1:**
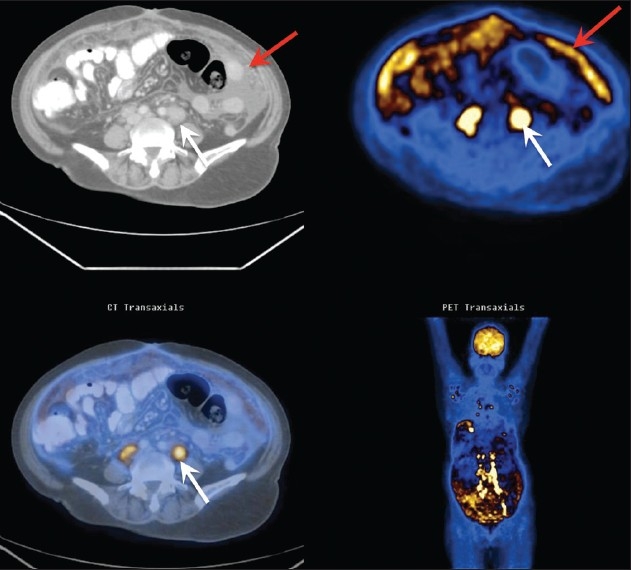
FDG-PET/CT demonstrates metabolically active retroperitoneal adenopathy (white arrows), ascites, metabolically active peritoneal deposits and extensive metabolically active omental thickening (red arrows)

A 56-year-old woman presented with ascites and lymphadenopathy, which were suggestive of metastases. An FDG PET/CT scan revealed [Figure 1] metabolically active, extensive lymphadenopathy in the axilla, mediastinum, abdomen, pelvis, and in the inguinal regions; there was also diffuse omental thickening with enhancement, a few faintly enhancing peritoneal soft tissues, right pleural effusion, and ascites. There was a maximum Standard Uptake Value (SUV) of 3.2 in the periportal, perigastric, peripancreatic, retrocaval, and retrocrural lymph nodes. The delayed PET/CT images demonstrated a slight increase in the SUV. A probable diagnosis of lymphoma was considered. Biopsy from a metabolically active retroperitoneal lymph node ruled out lymphoma and confirmed tuberculosis.

## Case 2

**Figure 2 (A, B):**
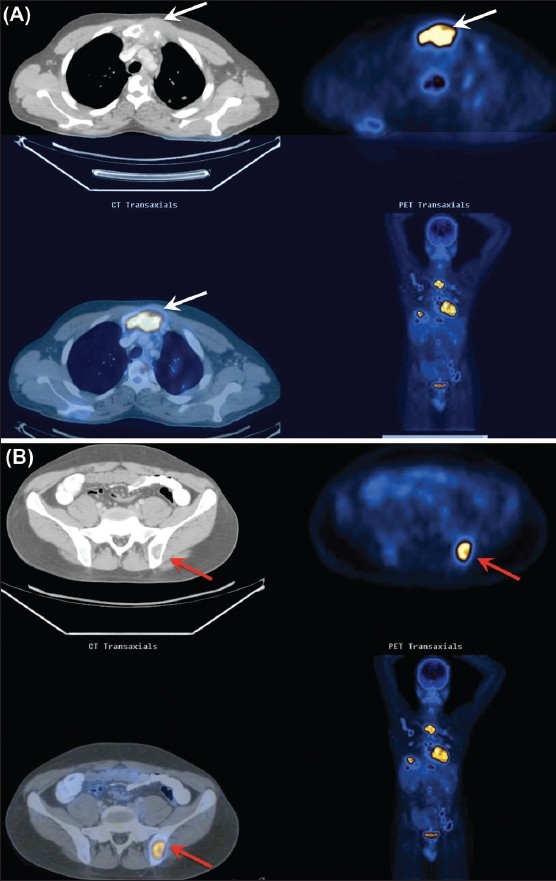
FDG-PET/CT of the chest (A) demonstrates metabolically active sternal (white arrows), vertebral body, and scapular lesions, as well as metabolically active left lung nodules. Biopsy from the sternal lesion showed tuberculosis. FDG-PET/CT (B) of the pelvis demonstrates a metabolically active osteolytic lesion in the left ilium (red arrows). Biopsy from this lesion showed tuberculosis as well

A 24-year-old male presented with mild diffuse skeletal pain. No other systemic symptoms were present. The patient was well built and nourished. An FDG PET/CT scan revealed [Figure 2] metabolically active, extensive skeletal osteolytic lesions in the calvarium, in the vertebral column at multiple levels, including the body and posterior elements and in the sternum, scapulae, ribs, and pelvic bones. A few sclerotic foci were also noted in the T4 and T5 vertebral bodies. No metabolically active lymphadenopathy was identified. A large metabolically active soft tissue mass was associated with the sternal lesion. A maximum SUV of 3.7 was seen in some of the skeletal lesions. Multiple metabolically active lung nodules were seen in both upper lobes and in the apical segment of the left lower lobe, with a maximum SUV of 1.5. On delayed images, there was significant increase in the SUV value of the majority of the lesions. A working diagnosis of skeletal metastases was considered. Biopsy from the sternal lesion ruled out malignancy and was indicative of tuberculosis. Biopsy from another site (left ilium) confirmed the diagnosis of tuberculosis.

Tuberculosis can have a varied presentation. There are reports of metabolically active breast masses with extensive axillary, cervical, and mediastinal lymphadenopathy that were initially mistaken for breast cancer with extensive lymph nodal metastasis but were later confirmed to be of tuberculous etiology.[1]

The above cases demonstrate the inadequacy of PET in the presence of tuberculosis. The associated diagnostic CT scan, though still not very specific, can be very useful in demonstrating the morphological details, particularly when used with intravenous contrast. Necrotic lymph nodes on contrast-enhanced CT scan (CECT), centrilobular lung nodules on high-resolution CT scan of the lungs, and soft tissue calcifications etc., can help in arriving at a confident diagnosis of tuberculosis. In high-prevalence geographic regions like India, tuberculous etiology should always be considered in the differential diagnoses and must be ruled out before a diagnosis of malignancy is made.

Newer and more specific radiotracers like positron-emitter labeled antituberculous drug molecules may help to differentiate tuberculosis from cancer and nontuberculous inflammatory processes in the future.[2]

## References

[1] Das CJ, Kumar R, Balakrishnan VB, Chawla M, Malhotra A (2008). Disseminated tuberculosis masquerading as metastatic breast carcinoma on PET-CT. Clin Nucl Med.

[2] Harkirat S, Anand SS, Indrajit IK, Dash AK (2008). Pictorial essay: PET/CT in tuberculosis. Indian J Radiol Imaging.

